# The sociology of artificial intelligence development and utilisation in Africa

**DOI:** 10.3389/fsoc.2026.1917558

**Published:** 2026-10-27

**Authors:** Ayodele Samuel Jegede, Olayinka Akanle, Olusegun Israel Olaniyan

**Affiliations:** 1 Department of Sociology, Faculty of the Social Sciences, University of Ibadan, Ibadan, Nigeria; 2 Department of Sociology, Faculty of Humanities University of Johannesburg, JohannesburgSouth Africa; 3 Department of Sociology, Ladoke Akintola University of Technology, Ogbomoso, Ogbomoso, Nigeria

**Keywords:** AI Africa, AI utilisation, artificial intelligence (AI), decolonial theory, sociology of AI

## Abstract

Artificial Intelligence (AI) is impacting societies’ social relations, institutions and social structures in definitive ways. Not many innovations have impacted human behaviours as significantly as AI. Deviating from the previous studies on AI that focuses on the potential implications of AI for everyday human society, this paper adopted secondary method of data collection, decolonial theory, and case study analysis to examine the sociological utilisation of AI in Africa. It provided answers to the fundamental sociological question of—why sociology of AI is needed in Africa? What forms of historical and social context shape AI’s incursion into Africa? Findings from this study revealed that while AI in Africa is driving positive innovations like financial inclusion, climate resilience, agriculture and food security, rapid diagnosis, education and language inclusion and job creation, it is also exacerbating technological asymmetry, expanding existing inequalities, strengthening unequal geopolitical power relations and perpetuating historical institutional dynamics on the continent.

## Introduction

Artificial intelligence (AI) is increasingly a social reality not only within African society but also a global reality that cannot be disputed. It is conditioning and being conditioned by belief systems, attitudes, constructions, interpretations, practices, and behaviours on the continent (Ade-Ibijola and Okonkwo, 2023). AI is creating, maintaining, and shaping how Africans perceive and interact with the world. Increasing aspects of Africa are being penetrated and impacted by AI (Kwarkye, 2025). These aspects include social institutions, languages, symbols, norms, laws and social structures that shape African human behaviours. As AI shapes innovation and governance, impacts economic structures, and drives social transformation, one pertinent point to note is that everyday life in Africa is being significantly affected by AI. While AI has the capacity to jumpstart and drive growth and development in Africa, for instance, through technology, finance, healthcare and education (Au and Fong, 2025; Mienye et al., 2024), the unique structure, power relations, social relations and historical contexts of Africa must be taken into account in the development and utilisation of AI. This is particularly true because technological innovations, such as AI, do not develop in a vacuum but within pre-existing social systems and structural conditions.

While scholars have held divergent views on the potential advantages of AI, its social implications and ethical considerations have also received widespread attention (Zhou, 2024). For the past few years, existing studies on AI have delved critically into the sociological conception and importance of AI (Mlynář et al., 2018; Davis and Sloane, 2026), relationship between AI tools usage and critical thinking (Gerlich, 2025), the role of sociology in enhancing AI integration in economic sectors (Constantinos, 2025), the typological readings of sociology of AI (Liu, 2021), and the role of sociological knowledge in AI language modelling and socialisation. Despite the volume of work on AI, a common trend across the identified studies is their predominant focus on the potential implications of AI for everyday human society. While the identified studies are worth acknowledging and imperative, there is still a dearth of studies on how AI’s development and utilisation reflect and reshape existing social structures within the context of African society. Scholars such as Joyce and Cruz (2024) have highlighted the sociological implications of AI for inequalities, power, and data justice. However, this study lacks an in-depth theoretical explanation and pays limited attention to the asymmetrical relationships inherent in the everyday utilisation of AI in Africa.

Africa presents a unique opportunity to unpack various social facts enshrouded in everyday AI utilisation, given decades of colonial and slave-trade experiences, bilingual cultural differences, and the slow pace of development (Nunn and Wantchekon, 2011). In addition, Africa presents a unique social, political, and economic reality that differs from the Global North and may not be fully understood from Eurocentric perspectives (Chumbow, 2009). Deviating from the conventional studies of sociology of AI, this article approaches issues surrounding AI from a decolonial perspective by raising an important sociological question: what happens when AI machines designed with systematised Western data structures encounter African informal social realities? What forms of African knowledge are excluded because they are not technologically readable? Whose knowledge becomes visible globally and whose knowledge becomes marginalised or underrepresented? Why is the sociology of AI essential in Africa? What forms of historical and social context shape AI’s incursion into Africa? What lessons can we learn from Kenya’s AI strategy and governance structure? By bridging the identified gap and addressing the question raised, the core contribution of this article lies in its exploration of the extent to which AI’s development and utilisation in Africa reflect and reshape existing social structures. It argues that, in order to harness the potential inclusive, ethically sound, and locally relevant benefits of AI, there is a need to understand its sociological relevance.

Given the scale and internal diversity of the African continent, this article does not claim uniform coverage of all national and regional experiences. Rather, it treats Nigeria, Kenya, and South Africa, the countries most represented in its document corpus, as illustrative cases through which broader continental dynamics of technological dependency, colonial legacy, and asymmetrical AI governance can be examined, while remaining attentive to the limits of generalising from these cases to the continent as a whole.

## Material and Methods

Rather than treating secondary text as unstructured background, this article is anchored purposively on diverse collection of secondary documents. This collection is structured along three documents’ types: (i) peer reviewed journal articles on artificial intelligence (AI), sociology of AI and African development; (ii) institutional and policy documents, principally the Kenya AI Strategy 2025–2030 (Kenya Ministry of Information, Communications and the Digital Economy, 2025) and the industry report Artificial Intelligence in Sub-Saharan Africa (Google, Sand Technologies and African Leadership University, 2025). The available data were sourced and downloaded from multiple platforms, including Google Search Engine, ResearchGate, Google Scholar, IEEE Xplore, and JSTOR. These databases were selected due to their extensive indexing of AI and sustainable development literature across diverse disciplines. To ensure that available downloaded studies align with the study focus, the search strategy involved using a combination of keywords such as “artificial intelligence,” “AI and digital divide”, “AI in Africa”, “Evolution of AI” and “Sociology of AI”.

Furthermore, a Critical Reliability Assessment (CRA) (also called Critical Appraisal Skills) was conducted on the downloaded articles to ensure the credibility of articles from reputable journal outlets and to filter out literature that does not align with the study’s focus. CRA is vital as it enables the researcher to “filter the grain from the chaff” for the purpose of making sense of methodologies adopted, achieve greater reliability and avoidance of redundance literature, and drawing viable conclusion that are crucial for the success of the research in a particular situation (Singh, 2013). This helps also ensure that only articles whose primary objective relates to AI, the Sociology of AI, and decolonisation were incorporated into the study. The inclusion criteria for selecting studies are those whose publication dates fall within the last 12 years (2012–2025). This publication timeline represents a period when there is huge proliferation of AI research in Africa (Kondo and Diwani, 2023). It also represents a period when IBM and other indigenous startups companies like InstaDeep open their African research lab in Nairobi, Kenya, and Tunisia, respectively, Google, Sand Technologies and African Leadership University (2025).

To achieve greater credibility and topical alignment, studies that focus primarily on technical advancements or computational aspects of AI without any relation to sustainable development or development in Africa are excluded. At the analyses level, filtered article were content analysed through narrative format. The form of analysis allows the researcher to document how each text, symbols, and meaning in the selected documents connect with each other (Zougris, 2025). Since the study involves the use of publicly available documents, it strictly upholds academic integrity and transparency by ensuring that all external ideas, statements, and data sourced from the literature are adequately acknowledged, cited, and referenced in accordance with best global ethical practices.

## Why sociology of AI in Africa

Strategically, the Sociology of AI aims to provide intellectual context and systems for the effective understanding of the history, currents, and dynamics of ways and manners that shape and affect human relations, human conditions, and behaviours (Au and Fong, 2025). This is particularly so in Africa. The Sociology of AI examines the subjectivities and objectivities of AI development and utilisation, and their implications for social relations and human behaviour, not only now but into the future (Liu, 2026; Liu, 2021). The Sociology of AI is thus key in Africa because development in Africa is shaped by both internal and global influences.

Globally, development stems from advances in technology, leading to economic and social transformation, which creates new ways of life and new methods of economic production and distribution of goods and services (Qureshi, 2019). A major incursion into society is Artificial Intelligence, which is now redefining the development agenda. Its implications for the African continent are not in doubt. It is driving development and shaping all aspects of life. Generally, Artificial Intelligence raises sociological questions of power, inequality, culture, knowledge, ethics, and agency despite its importance (Colther and Doussoulin, 2024). Throughout the world, societies are organised specifically to meet people’s needs. The structure of societies shapes people’s lives and ensures their access to basic needs. The adaptation of AI will no doubt influence the existing social structure in ways that are not yet clear.

## The sociology of AI from Decolonial perspective

Decolonial theory helps us understand that diverse worlds and alternative ways of worlding coexist (Blaser, 2010; Manning, 2026). Emanating from Latin America, decolonial theorists posit that the world and what is considered as knowledge are created based on a European ontological perception of modernity, which tends to eradicate the worldviews, ideas, institutions, and knowledge of those in the less privileged society (Dussel and Ibarra-Colado, 2006; Escobar, 2007; Mignolo, 2007). Quijano (2007) expands on this within the framework of the coloniality of power, which he argues is the continuous categorisation and discriminatory discourse manifested in the social and economic structures of modern postcolonial societies. According to Quijano (2007), this form of relations can be found within the four domains of power and control—control of economy (land appropriation, exploitation of labour, control of natural resources), control of authority (government, institution, army), control of gender and sexuality (family, identity, heteropatriarchy), and control of subjectivity and knowledge (epistemology, education, formation of subjectivity).

Bertolt (2018) argues that the coloniality of power is the “theft of history” and understanding its manifestation makes it possible to decipher the world as racialised, colonial, capitalist, patriarchal, hierarchical, heteronormative, neoliberal, hegemonic and Euro-American. A decolonial theoretical perspective is crucial for understanding the realities and trajectories of AI in the context of its development and utilization in Africa (Mbah and Jarvis, 2026). Across the micro (individual identity, behaviour, and decision-making processes), meso (community, institutional, and organizational levels), and macro (continental and global inequalities) levels, AI exacts and exercises agency (United Nations Development Programme (UNDP), 2025). AI accentuates global and social inequalities and systemic relational hierarchies that have huge implications for development and utilization in the short and long run (United Nations Development Programme (UNDP), 2025). Because AI needs substantial inputs like data and human skills/capabilities that are unevenly distributed and shape availability in Africa, AI tends to reproduce and accentuate established social inequalities, such as the digital divide and racial and economic inequalities (Joyce et al., 2021).

Decolonial perspective is important in understanding Africa’s unique situation. This is because AI is both a contemporary technological reality and a social construction that ultimately reflects historical patterns and trajectories of Africa (Kitonga, 2025). AI is largely a rehash of Africa’s development experiences, which continue to be shaped by colonial legacies (Ooko, 2025). There are asymmetrical relationships in the development and utilization of AI between Africa in the global south and developed countries of the global north. These continue to demonstrate and perpetuate historical domination, technological overpowering and epistemic exclusion of Africa. There is thus a need to decolonize AI by decentring AI in technological inputs and constructions. There is a need to factor in more organic, diverse knowledge apparatuses and content that include context, such as Africa, into the AI development and utilization ecosystem (Ooko, 2025). AI approaches and systems that ignore Africa’s colonial trajectories cannot be considered real and ready (Kitonga, 2025; Ooko, 2025). It is therefore not surprising that Africa is already being left behind in the AI journey. This is usually the case in Africa when it comes to innovation and transformation. The current reality is that AI is increasingly expanding and reconfiguring the unbalanced technological power relations against Africa. This will continue to be so except that AI is decolonized (see Salami, 2024).

The AI, as a tool for perpetuating colonial systems, becomes evident when Africa is viewed through the lens of not being sufficiently competitive in the global economic, political, and technological space (Brown et al., 2026). For instance, most existing and strategic technological infrastructures, skills, tools (software and hardware), demands, markets, and overall knowledge production are determined and moderated by external agencies, interests, actors, and institutions. This has always been the case historically since the colonial era. AI operates on global knowledge and interests, and Africa has not been effectively integrated from the outset. The development and utilization of AI as it affects Africa is inseparable from the continent’s structural conditions and colonial history (Brown et al., 2026). Africa’s post-colonial AI formations and conditions are manifestations of a deeper-seated colonial political economy and uneven grafting into the global technology-capitalist economy, often driven by dependency. Even in a few African countries, like Kenya, Nigeria, and South Africa, where there is increasing AI research and capabilities, their research, funding, demands, and capabilities are driven from outside Africa (Ayesu, 2026). This still demonstrates huge dependence on global dictates from outside the continent. Thus, the development and utilization of AI show significant imbalances and disparities between Africa and the global north, which is the epicentre of AI development and utilization.

## Evolution of AI: structural context, relevance and sociological gaps

The evolution of AI is a complex task enmeshed in different phases of human technological advancement. Though the idea of transforming inanimate objects into intelligent beings has been conceived by mankind and documented in Ancient Greeks, Chinese and Egyptian mythology, and classical philosophers thought of the human symbolic system (Mijwel, 2015), the foundation of AI started between the 1950s and 1970s with Alan Turing’s milestone paper titled “Computing Machinery and Intelligence (Delipetrev et al., 2020). Turing, in his paper, asked a fundamental question: “Can Machines Think?” and proposed an imitation game (known as the Turing test) to determine whether a machine can engage in a meaningful conversation similar to that of a human (Delipetrev et al., 2020). With this idea, evidence abounds that the Turing test was the first computer experiment to determine the degree of machine intelligence.

Following this impeccable test, it is worth noting that the AI field was formally created at the Dartmouth Conference of 1956. The Dartmouth Conference not only marked the emergence of AI but also saw McCarthy coin the term “artificial intelligence” (Delipetrev et al., 2020). The primary idea of this conference was “every aspect of any other feature of learning or intelligence should be accurately described so that a machine can simulate it” (Russell and Norvig, 2021). While people were excited that computers were providing solutions to problems like humans, it is also worth noting that the 1980s witnessed a paradigm shift in AI. The 1980s were characterised by symbolic AI and expert systems, also known as knowledge-based systems. The underlying idea was to harness human expert knowledge in digital form and distribute it as a program across many Personal Computers (PCs; Russell and Norvig, 2021).

These expert systems comprise two components—the knowledge base, which refers to the collection of facts, rules, and relationships on a specific domain; and the inference engine, which describes the processes of manipulating and combining symbols. During this period, companies began creating expert systems and offering software packages called “inference engines,” along with related knowledge, to several organisations and customers. While this period marked several important AI breakthroughs, such as MYCIN, SHRDLU, PROLOG, the Stanford Cart, Hopfield net, and ID3, these tools and frameworks lacked adequate expressive power to capture the breadth of expert knowledge and behaviour needed to achieve satisfactory performance (Delipetrev et al., 2020). Leveraging the anomalies of the previous AI built, the period from the 1990s onward witnessed the era of machine learning and deep learning systems. In the 1990s–2010s, AI had addressed some of the most difficult problems of human, providing solutions that were found to be valuable in diverse application domains including data mining, industrial robotics, logistics, business intelligence, banking software, medical diagnosis, recommendation systems, and search engines (Delipetrev et al., 2020).

This period also marked the era when many AI scientists began to create and use more complex mathematical language and economic tools to solve problems that could not be addressed in the previous era. This milestone provides an opportunity for greater collaboration with established fields and also makes AI a more rigorous scientific discipline (Delipetrev et al., 2020). While the Global North countries have witnessed huge proliferation of AI usage for more than three decades, a joint report published by Google, Sand Technologies, and African Leadership University in the year 2025 revealed that the roots of AI in Sub-Saharan Africa dated back to the early 2000s when academic institutions started to establish research programs focused on computational sciences (Google, Sand Technologies and African Leadership University, 2025). Notable initiatives during this period include the establishment of AI research labs and a high rate of international collaboration. During this period, institutions such as the African Institute for Mathematical Sciences (AIMS), established in 2003, began offering programs in machine learning and data science. This laid a stronger foundation and serves as a stepping stone for future AI research and education across the continent.

Furthermore, stronger collaboration with global technological companies also aided knowledge transfer and capacity building. For instance, the launch of the IBM Research Lab in Nairobi in 2013 marked a significant milestone investment in indigenous AI capabilities (Google, Sand Technologies and African Leadership University, 2025). The growth of AI has revolutionised African ecosystems, with more startups, companies, and government initiatives emerging to boost the continent’s development. Available evidence abounds that the year 2023 saw the emergence of over 2,400 AI-focused companies and tech hubs in countries such as South Africa, Nigeria, and Kenya. Also, several African governments have recognised the role of AI in promoting economic growth. For instance, South Africa created the Presidential Commission on the Fourth Industrial Revolution to establish policies that promote AI adoption. In a similar vein, Kenya has also taken a bold step by establishing a task force to promote the effective use of AI (Google, Sand Technologies and African Leadership University, 2025).

With this acceptance rate of AI in Africa, there is no doubt that AI has shown promise in the various fields of human endeavour, particularly in the area of health, agriculture, education, finance, and public services (Aijaz, N. et al., 2025; Ajayi et al., 2018; Villanueva-Miranda et al., 2025). Studies have also shown how AI’s application is revolutionising infectious disease diagnosis and monitoring, predicting outbreaks, and expert systems in patient care (Villanueva-Miranda et al., 2025). This transformation is not only in Nigeria but also elsewhere, thereby depicting the global influence of AI (Kehinde et al., 2024). For instance, in Nigeria, the “HelpMum ADVISER framework” (AI-Driven Vaccination Intervention Optimiser) has been deployed to more than 13,000 families in Oyo State to optimise vaccination programmes. This demonstrates how AI can be used in public health interventions to achieve real social impact particularly on the need to guide government agencies to increase vaccine uptake in resource-constrained environments (Abdu-Raheem and Musa, 2025). Also, AI has the potential to improve early diagnosis, precision medicine, and remote monitoring in paediatrics. However, inadequate infrastructure and weak ethical and regulatory services still pose challenges to this potential (Okwukwu et al., 2025; Olawade et al., 2025) resulting in important sociological gaps.

Despite the promise AI holds, several sociological gaps emerge relative to knowledge gaps and the digital divide. Studies among healthcare students and professionals in Nigeria reported important knowledge gaps. For instance, a quantitative study on artificial intelligence readiness among healthcare students at Obafemi Awolowo University to determine students’ self-perceived and actual knowledge of AI in healthcare reveal that while 60% of respondents believed they possessed high levels of AI skills, objective assessment revealed that approximately 92% of respondents demonstrated limited/low skills levels, with only 0.4% achieving high knowledge scores (Olawade et al., 2025). Findings from this study further reveal concerning performance, with only 12% of respondents correctly defining machine learning, 7.5% understanding supervised and unsupervised learning, and 10.2% grasping Internet of Things concepts. The lack of understanding of fundamental concepts such as training data bias, model validation, and algorithmic transparency is particularly problematic, as these are essential for safe and effective AI implementation in healthcare (Olawade et al., 2025).

Beyond the stated knowledge gap, the rise of digital inequalities has also become a notable sociological challenge associated with AI. Aligning with the positions of Bigman et al. (2023); Johnson (2021), and Wang et al. (2022), digital inequalities do not arise solely from the introduction or termination of technology. However, rather than erupt due to language differences, cultural differences, and algorithmic sentiment that determine the degree of interaction learners of AI tools engage in. While cultural sentiment refers to individual feelings of ethnocentrism, algorithmic bias occurs when a computer system systematically produces unfair outcomes, often due to embedding human ethnocentric biases in the system’s configuration and training data (Fitas et al., 2025; Seaver, 2017).

This is reflected in the technology’s language configuration, which is predominantly designed in English and perhaps other major European languages, with little accommodation for multilingual or indigenous and cultural languages (Matjie et al., 2026). This linguistic and cultural sentiment reduces the relevance and teaching and learning valency of AI systems for different populations, thereby creating marginalisation through design rather than access alone (Mac Fadden et al., 2024). Having discussed the inherent sociological problems of AI, the pertinent question that begs an answer is how African countries are navigating the sociological dimensions that accompany AI. In answering this question, a critical look at Kenya’s AI strategy and institutional governance will help provide a deeper analysis of the phenomenon under investigation.

Before delving deeper into this analysis, it is worth noting that Kenya was position in this article as an aspirant and adopter (“aspires to become”, “regional leader”, “forerunner”) rather than as an originator of AI paradigms, even as it calls for “local talent” and “indigenous knowledge.” Responsible and ethical usage of AI are defined largely through alignment with “global human rights standards” rather than through Kenyan or African normative frameworks, so the discourses on Kenya AI strategy an institutional governance reproduces the asymmetry power relationship the article decolonial framing seeks to critique. That is, sovereignty is asserted at the level of talent and data, but legitimacy is still anchored externally.

## A case study: Kenya’s AI strategy and institutional governance

Kenya offers a useful case study for exploring how an African country is navigating these sociological dimensions. Kenya aspires to become a global force in the African AI landscape by creating a comprehensive, diverse, and inclusive AI ecosystem (Kenya Ministry of Information, Communications and the Digital Economy, 2025). This AI governance strategy focuses on creating an enabling environment for the responsible development of high-quality AI applications that leverage indigenous information and talent, ensuring safety, accountability, and alignment with global human rights standards. Kenya’s AI strategy aims to promote economic growth, public-sector efficiency, international competitiveness, protection against the harmful impacts of externally developed AI solutions, and employment creation and skills development (Kenya Ministry of Information, Communications and the Digital Economy, 2025). The strategy envisions Kenya as a forerunner in quality AI innovation and development, and in establishing innovative and standardised solutions that cater to the needs of local and diverse communities (Kenya Ministry of Information, Communications and the Digital Economy, 2025).

The vision is to be a regional leader in AI research and development, innovation and commercialisation for inclusive socio-economic development. To achieve this, Kenya aims to leverage local talent, indigenous knowledge, and skills to reconfigure vital sectors to foster inclusive economic growth and development. Also, Kenya aims to achieve this by upholding ethical and responsible AI by setting clear guidelines that balance innovation with ethical considerations, ensuring AI is created and channelled safely, responsibly, and inclusively (Kenya Ministry of Information, Communications and the Digital Economy, 2025). By prioritising data sovereignty and ethical AI practices, Kenya aims to create a technological future through effective and efficient collaboration with local and international stakeholders that helps to define and embrace best practices in AI adoption, global competitiveness, and safety ecosystem (Kenya Ministry of Information, Communications and the Digital Economy, 2025).

To achieve this technological progress, Kenya AI is anchored on the following principles. These include the need to promote an inclusive and non-discriminatory approach in the development and deployment of AI, encourage multi-stakeholder participation, transparency and accountability, cultural preservation and contextualisation, environmental sustainability, economic benefit and self-sufficiency, and prioritisation of local talent and resources (Kenya Ministry of Information, Communications and the Digital Economy, 2025). Kenya’s overall AI strength lies in the existing diverse and vibrant local innovation ecosystem, young digital-ready AI workforce, foundational legal framework for ethical AI development, existing digital infrastructure, political will and strong government support for the adoption of AI, and homegrown companies and regional tech hub (Kenya Ministry of Information, Communications and the Digital Economy, 2025).

Despite the strength and potential progress in the field of AI, Kenya still faces significant sociological challenges. Specifically, Kenya faces a significant shortage of AI experts capable of training contextually relevant AI models. While existing legal frameworks like Data Protection, Computer Misuse and Cybercrime, Intellectual Property, and Consumer Acts have been established to provide specific guidance, another major bottleneck is that Kenya lacks a holistic and regulatory framework and policy specifically targeted toward AI (Kenya Ministry of Information, Communications and the Digital Economy, 2025). Furthermore, despite the availability of digital infrastructure, Kenya still struggles with insufficient `ability and poor data quality, both of which are essential for achieving an effective AI system (Kenya Ministry of Information, Communications and the Digital Economy, 2025).

Expounding on the above view, it is worth noting that while Kenya has made significant progress in digital infrastructure, data accessibility, and computing power for AI applications, there remain major challenges that limit the scalability of AI-driven solutions nationwide. Similarly, equity and inclusion in AI development and gaps in public AI/digital literacy are major challenges confronting the overall utilisation of AI in the country. This not only exacerbates the digital divide between urban and rural areas and among marginalised communities but also hinders the adoption of AI-driven solutions and potentially increases socioeconomic inequalities in the country (Kenya Ministry of Information, Communications and the Digital Economy, 2025).

## Conclusion and recommendations

Artificial Intelligence in Africa carries the potential to address some of the continent’s long-standing development and social challenges. However, its evolution is not merely technological but deeply social. The terms of its development matter: who participates, how benefits are distributed, which values guide design, and which harms are mitigated. Only through a sociologically informed approach that considers history, power, culture, inequality, and agency can AI lead to equitable and sustainable transformation in Africa. For Africa to benefit from AI’s potential, scholars, policymakers, developers, and civil society must work together to build an AI ecosystem that is not only efficient and innovative but also just, inclusive, and rooted in the values of African societies. This is very important because the development and utilisation of AI are in themselves deep cultural, social, historical, economic and political realities. Through effective co-design and participatory development, it will ensure that AI systems are created with the active involvement of communities, especially marginalised populations, civil society groups, and indigenous knowledge holders. For example, farmers can help design AI-based climate advisory tools suited to local conditions, while persons with disabilities can guide the development of accessible health applications.

Indigenous communities may also contribute traditional knowledge to environmental monitoring systems. This inclusive approach will help to promote fairness, cultural relevance, trust, and accountability in AI deployment. Also, context-aware and flexible regulatory frameworks are essential for responsible AI governance. Legal and policy instruments should anticipate emerging risks, adapt to technological changes, and include ethical safeguards. For example, data protection laws can ensure patient privacy in digital health systems, while clear liability policies can address harm caused by autonomous technologies. Flexible regulations also support innovation by allowing periodic review and adjustment. Such frameworks promote accountability, data sovereignty, transparency, trust, and equitable use of AI across different social and cultural contexts. Furthermore, capacity building is essential for sustainable and inclusive AI development. Governments and institutions should invest in human capital through education, digital skills training, and AI literacy programmes for medical practitioners, students, researchers, and end-users. Strengthening infrastructure such as broadband internet, electricity supply, and data centres will support innovation and accessibility. For example, training healthcare workers in AI-assisted diagnostics can improve service delivery, while research institutions can drive the development of local AI solutions. These investments enhance knowledge, reduce digital inequality, and promote effective, ethical AI adoption.

Another way of promoting effective utilisation of AI in Africa is through ethical pluralism. This help to encourages AI governance models that reflect diverse cultural and philosophical perspectives rather than relying solely on Western ethical frameworks. Indigenous philosophies such as Ubuntu, which emphasize community, interconnectedness, and shared humanity, can serve as normative foundations for AI governance. For example, communal data trusts may promote collective ownership of data, while relational accountability ensures technologies consider social relationships and responsibilities. Ecological sensibility and intersectional fairness also encourage sustainable, inclusive, and equitable AI systems that respect both human and environmental wellbeing across diverse communities. By bridging the inherent digital and social divides created by AI, it will help to promote an inclusive technological development that include rural populations, women, speakers of underrepresented languages, and people in remote areas in AI development and deployment.

This will make AI more accessible, culturally relevant, affordable, and responsive to local needs. For example, healthcare chatbots designed in local languages can improve access to health information in rural communities, while mobile-based agricultural AI tools can support women farmers. Ensuring accountability and inclusiveness in these contexts helps reduce inequality, promote participation, and expand the benefits of AI to underserved populations. Finally, empirical research and evaluation are essential for understanding how AI systems function in real-world African contexts. This requires grounded socio-technical and ethnographic studies that examine lived experiences, trust levels, effectiveness, and unintended consequences of AI deployment. For example, field studies of AI-driven health tools can reveal how users interpret recommendations, while community-based research can assess barriers to adoption in rural areas. Such evidence-based insights help improve design, ensure cultural relevance, and guide responsible, context-sensitive AI development and policy decisions.

From an AI and decolonial perspective, it is imperative to decentre narratives, development and utilisation of AI. It is vital to challenge Western hegemony and dominance in AI. This can be done by promoting, institutionalising, and narrating realities and experiences grounded in African norms, values, innovations, systems, structures, institutions, and cultures. Attention must therefore be paid to best practices, originality, and innovation, no matter how small. Hence, it is necessary to ensure African participation in AI development, utilisation and governance. This should include local narratives, culture, and indigenous knowledge systems relevant to AI development and utilisation. Africa should not be seen as mere consumers of AI but rather as necessary AI partners. This needs to be reflected in the development and implementation of AI ethical frameworks, as well as in their development, utilisation, and distribution.
